# Income Disparity and Risk of Death: The Importance of Health Behaviors and Other Mediating Factors

**DOI:** 10.1371/journal.pone.0049929

**Published:** 2012-11-19

**Authors:** Soghra Jarvandi, Yan Yan, Mario Schootman

**Affiliations:** 1 Division of Health Behavior Research, Washington University School of Medicine, Saint Louis, Missouri, United States of America; 2 Division of Public Health Sciences, Department of Surgery, Washington University School of Medicine, Saint Louis, Missouri, United States of America; University of Ottawa, Canada

## Abstract

**Background:**

Income disparities in mortality are profound in the United States, but reasons for this remain largely unexplained. The objective of this study was to assess the effects of health behaviors, and other mediating pathways, separately and simultaneously, including health insurance, health status, and inflammation, in the association between income and mortality.

**Methods:**

This study used data from 9925 individuals aged 20 years or older who participated in the 1999–2004 National Health and Nutrition Examination Survey (NHANES) and were followed up through December 31, 2006 for mortality. The outcome measures were all-cause and CVD/diabetes mortality. During follow-up 505 persons died, including 196 deaths due to CVD or diabetes.

**Results:**

After adjusting for age, sex, education, and race/ethnicity, risk of death was higher in low-income than high-income group for both all-cause mortality (Hazard ratio [HR], 1.98; 95% confidence interval [CI]: 1.37, 2.85) and cardiovascular disease (CVD)/diabetes mortality (HR, 3.68; 95% CI: 1.64, 8.27). The combination of the four pathways attenuated 58% of the association between income and all-cause mortality and 35% of that of CVD/diabetes mortality. Health behaviors attenuated the risk of all-cause and CVD/diabetes mortality by 30% and 21%, respectively, in the low-income group. Health status attenuated 39% of all-cause mortality and 18% of CVD/diabetes mortality, whereas, health insurance and inflammation accounted for only a small portion of the income-associated mortality (≤6%).

**Conclusion:**

Excess mortality associated with lower income can be largely accounted for by poor health status and unhealthy behaviors. Future studies should address behavioral modification, as well as possible strategies to improve health status in low-income people.

## Introduction

Income disparities in health outcomes remain profound despite national and local efforts [1]. According to a study in the United States in 1998, people with low income, i.e. annual household income <$10,000, were about three times more likely to die than those with higher income (≥ $30,000) [2]. The impact of low income on mortality is likely transmitted through several potential mediating pathways that influence an individual’s health [3]. Although numerous studies have examined the association between socioeconomic factors and health [4]–[8], the mechanisms are yet to be explored. Two early interpretations for health disparities include material deprivation and psychosocial mechanisms. While some researchers highlighted the importance of material resources, such as food and access to services [4], others emphasized psychosocial conditions such as depression, insecurity and stress [5]–[8]. Further, some studies have moved beyond associations and quantified the extent of mediating effects of individual pathways [2], [9], [10]. Expanding the examination to multiple mediators will allow for quantifying the relative contribution of different mediators, thus help planning towards health equity, a goal of the Healthy People 2020 in the United States [11].

Several key risk factors of mortality are more common among individuals with low income. These include, first, unhealthy behaviors, such as consumption of a low-quality diet, physical inactivity, tobacco use, and heavy alcohol use [12], second, lack of health insurance, a key indicator of access to medical care in the United States [13], and third, worse health status, including disability and poor perceived health [14]. In addition, chronic systemic inflammation, a biological predictor of all-cause and cardiovascular mortality, is increased, independent of health behaviors, among people with low income [15], [16]. Although the association between low income and risk factors of mortality has been well documented, little is known about the extent to which these risk factors, each and in combination, mediate the association between income and mortality. Previous work has shown that health behaviors mediated the association between low socioeconomic status and mortality only by 12% in a US study [2] and by 42% in a European study, with single assessment, and by 72% with repeated assessments [9], indicating the importance of examining other possible mediators besides health behaviors in order to have a more comprehensive understanding of income-associated mortality.

Previous studies used various measures of income to examine different hypotheses about the negative relation between income and health outcomes [17]. While ecological-level studies explained the risk of death associated with income inequality, for example at the state level [18], others used the individual-level measures [2]. An alternative approach, including both individual- and ecological-level measures of income, suggests that individual income, rather than income inequality, may be a better determinant of mortality [19].

Therefore, this study investigated the mediating effects of four pathways, separately and simultaneously, on the association between individual income and mortality, using data from a nationally representative sample of US civilians. Specifically, objectives of this study were to estimate the extent to which health behaviors, access to medical care, health status and systemic inflammation accounted for any observed association between low income and mortality.

## Methods

### Study Population

This study used data from the National Health and Nutrition Examination Survey (NHANES) collected in 1999 through 2004 [20]. NHANES is an annual, cross-sectional survey that collects data from a nationally representative sample of the non-institutionalized U.S population. The survey includes household interviews, physical examinations and blood sampling. The present study was limited to respondents who were 20 years or older (the age group that was eligible to answer all survey questions, including alcohol use), and for whom data on all the variables were available.

### Mortality

Information about deaths was obtained from the NHANES (1999–2004) Linked Mortality File, which was created by the National Center for Health Statistics (NCHS) by linking the NHANES data to the National Death Index (NDI). The public-use version of the linked data includes the mortality status of all respondents 18 years and older until December 31, 2006, as well as person-month follow-up, and underlying cause of death [20]. Two types of mortality outcomes were used: all-cause mortality and deaths caused by cardio-metabolic diseases including cardiovascular disease (CVD) and diabetes, both of which account for a large proportion of the socioeconomic disparity in mortality [21]. Deaths from CVD/diabetes were identified based on the *International Statistical Classification of Diseases, 10^th^ Revision* (ICD-10) (ICD-10 codes 100–178 180–199 E10–E14).

### Income

Respondents completed questions on annual total family income and family size. NHANES, then, calculated a Poverty Income Ratio (PIR), the ratio of family income to the poverty threshold for the family size in the year of the interview, using tables on poverty that are published yearly by the U.S. Census Bureau. In contrast to family income, the PIR is an inflation-adjusted, and thus relatively stable, measure for analysis across years. PIR was categorized, according to NHANES guidelines [22], into low income (PIR ≤1.30: the federal cutoff point for eligibility for the Food Stamp Program), intermediate income (1.30<PIR ≤3.50), and high income (PIR>3.50).

### Potential Mediators

Health behaviors include diet quality, physical activity, alcohol use, body mass index (BMI), and cigarette smoking. Diet quality was assessed by the Healthy Eating Index-2005 (HEI-05), calculated from the reported dietary data. The HEI-05 ranges from 0 to 100, with higher scores indicating greater compliance with *Dietary Guidelines for Americans, 2005* [23]. Physical activity was measured by questions about leisure-time activity during the past month. For respondents who reported engaging in moderate/vigorous-intensity activity, NHANES assigned metabolic equivalent of task (MET) scores using the reported frequency, duration and intensity of the activity [20]. For those who did not report any moderate/vigorous-intensity activity, a score of 0 was assigned. Using total MET-hours per week scores, respondents were categorized, similar to a previous report [24], as: inactive (0), somewhat active (>0 to <9) and active (≥9). BMI, calculated from measured weight and height as kg/m^2^, was categorized as underweight (<18.5), normal range (18.5–24.9), pre-obese (25.0–29.9) obese class I-II (30.0–39.9) and obese class III (≥40.0). Smoking status was self reported and defined as current smoker versus non−/ex-smoker. Regarding alcohol use, respondents were classified as non-drinkers (consumed no alcohol over the previous year or <12 alcoholic drinks in any one year), moderate drinkers (≤2 drinks/day for men and ≤1 drinks/day for women, as defined by the U.S. Dietary Guidelines [23]), and heavy drinkers (>2 drinks/day for men and >1 drinks/day for women).

Health status at the time of the interview was assessed by self-report and included: self-rated health, and disability. Self-assessment of health, including self-rated health and functional disability are considered to be valid and useful indicators of health status [25], [26]. Participants were asked to rate their general health condition on a scale from 1–5; 1: excellent, 2: very good, 3: good, 4: fair, 5: poor. Following common practice, the answers were dichotomized as good health (1, 2, or 3), and poor/fair health (4 or 5). Disability was defined based on 19 activities involving 5 major domains: activities of daily living (ADL), instrumental activities of daily living (IADL), leisure and social activities (LSA), activities for lower extremity mobility (LEM), and general physical activities (GPA) [27]. Participants that reported to have any difficulty in at least one activity in a domain were considered to have a disability in that domain. Self-reported current health insurance coverage was used as an indicator of access to medical care. C-reactive protein (CRP), a marker of systemic inflammation, was measured in NHANES using a highly sensitive assay technique, using latex-enhanced nephelometry. Details about the laboratory procedures are reported elsewhere [28]. Higher values indicate more inflammation. CRP was categorized as <1 mg/L, 1–3 mg/L, >3–10 mg/L, and >10 mg/L [29]. Age, sex, race/ethnicity and education were assessed by self-report. Education was categorized as: <high school, high school graduate/some college, and ≥ college graduate.

### Statistical Analysis

**Figure 1:**
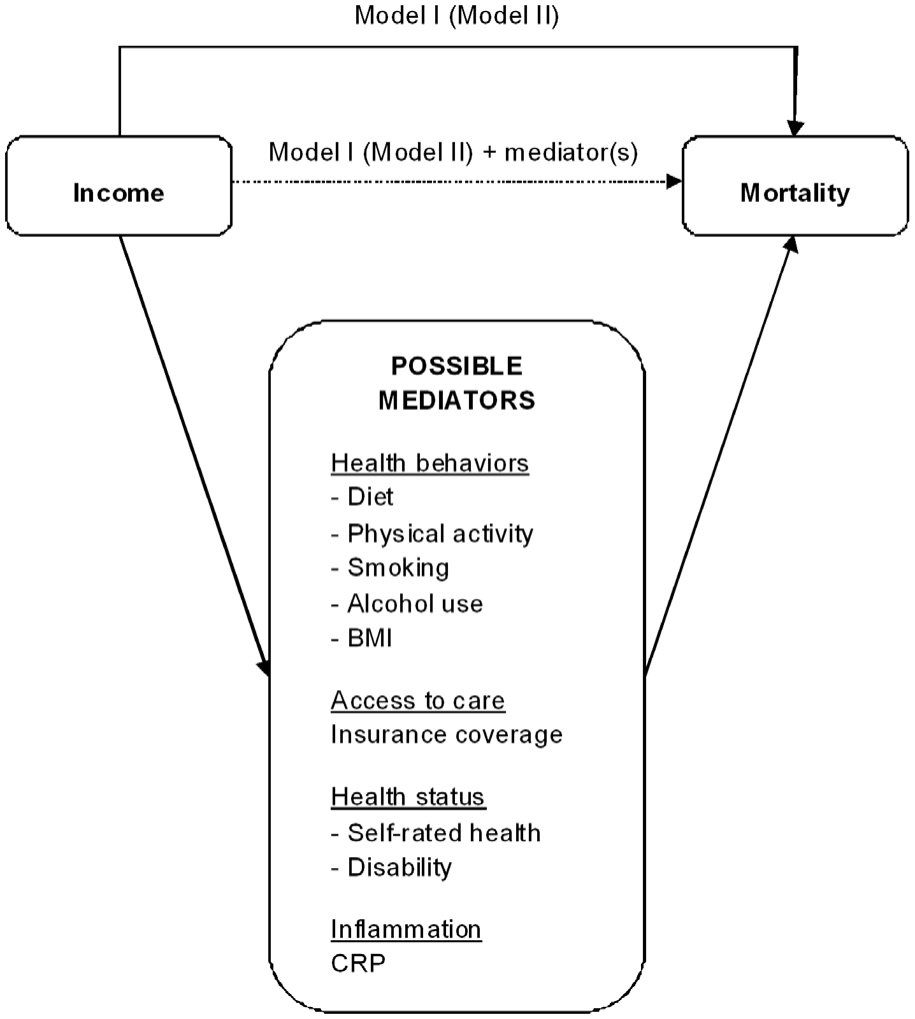
Schematic Diagram of Some Pathways That Mediate the Association Between Income and Mortality. Mediating effect was Calculated as: (β _Model I –_ β _Model I+mediator(s)_) × 100/β _Model I_ for all-cause mortality (β _Model II -_ β _Model II+mediator(s)_) × 100/β _Model II_ for CVD/diabetes mortality.

All statistical analyses were performed using SAS 9.2 proc survey procedures (SAS Institute, Cary, NC). Data from NHANES cycles 1999–2000, 2001–2002 and 2003–2004 were combined. In all analyses sampling design and weights were used to account for the complex sampling design in NHANES, thereby generating nationally representative estimates. Differences between the income groups were assessed by χ^2^ tests. Cox proportional hazards analyses were used to estimate the hazard ratio (HR) and corresponding 95% confidence intervals (95% CI) for the association between income category (low, intermediate, and high) and either all-cause mortality (Model I) or CVD/diabetes mortality (Model II), adjusted for demographic covariates (age, sex, and race/ethnicity). To test the mediating effect, the mediators were added, separately and simultaneously, to Model I (for all-cause mortality) and Model II (for CVD/diabetes mortality) (Figure 1). The extent of each mediating effect was calculated similar to a recent report [9]:

(β _Model I -_ β _Model I+mediator(s)_) × 100/β _Model I_ (all-cause mortality).

(β _Model II -_ β _Model II+mediator(s)_) × 100/β _Model II_ (CVD/diabetes mortality).

Additionally, 95% confidence intervals were constructed for the estimates of the mediating effects, using bootstrapping re-sampling technique [30].

Because health status and health behaviors are interrelated [31], the overlap and independent mediating effects of these pathways were also calculated. For this, the combined effect of health behaviors and health status was calculated by adding these two pathways in Model I and Model II. Then the combined effect was subtracted from the sum of two individual effects to get the overlap between the two pathways. Finally, the independent effect was calculated for each pathway by subtracting the overlap from the individual effect.

In addition to income, hazard ratios of mortality were estimated for education in the total population, also stratified by gender and race/ethnicity.

### Sensitivity Analyses

To evaluate whether a possible reverse causation between health and income, i.e. that low income caused by poor health, could affect the results, we conducted a sensitivity analysis excluding participants who were unemployed because of health reasons. Unemployment was define as reporting “looking for work” or “not working at a job/business” in the week before the interview. Among unemployed individuals, those who reported “unable to work for health reasons” or “disabled” were considered unemployed because of health reasons.

In addition, to examine the impact of mediating pathways among different age groups, age-stratified models were used, using three age groups: 20–44 years, 45–64 years, and 65 years and older.

**Table 1:** Baseline characteristics by income groups.

	Low income	Medium Income	High Income	*P* ^a^
	PIR ≤1.30	1.3 <PIR <3.5	PIR ≥ 3.50	
	N = 2617	N = 3855	N = 3453	
**Demographic**				
Age, mean (SE)	42.73 (0.46)	45.75 (0.42)	46.15 (36)	<.0001
Sex				<.0001
Female	1440 (55)	1986 (52)	1693 (49)	
Male	1177 (45)	1869 (48)	1760 (51)	
Race/ethnicity				<.0001
Non-Hispanic Black	555 (21)	744 (19)	479 (14)	
Mexican American	884 (34)	965 (25)	381 (11)	
Other	259 (10)	258 (7)	177 (5)	
Non-Hispanic White	919 (35)	1888 (49)	2416 (70)	
Education				<.0001
<High school	1430 (55)	1181 (31)	300 (9)	
High school/some college	1060 (41)	2183 (57)	1759 (51)	
≥ College graduate	127 (5)	491 (13)	1394 (40)	
**Health behaviors**				
BMI (kg/m^2^)				<.0001
≥40.0	151 (6)	182 (5)	135 (4)	
30.0–39.9	733 (28)	1060 (27)	892 (26)	
25.0–29.9	891 (34)	1421 (37)	1289 (37)	
18.5–24.9	797 (30)	1134 (29)	1094 (32)	
<18.5	45 (2)	58 (2)	43 (1)	
MET-hours/week of leisure-time physical activity				<.0001
Inactive (0)	1450 (55)	1701 (44)	893 (26)	
Somewhat active (>0 to <9)	698 (27)	1159 (30)	1130 (33)	
Active (≥9)	469 (18)	995 (26)	1430 (41)	
Diet (HEI-2005 quintiles)				<.0001
Q1	615 (24)	784 (20)	585 (17)	
Q2	557 (21)	774 (20)	645 (19)	
Q3	525 (20)	806 (21)	664 (19)	
Q4	508 (19)	774 (20)	711 (21)	
Q5	412 (16)	717 (19)	848 (24)	
Current smoking				<.0001
Yes	782 (30)	866 (22)	532 (15)	
No	1835 (70)	2989 (78)	2921 (85)	
Alcohol use				<.0001
Non-drinker	1347 (51)	1702 (44)	1035 (30)	
Moderate drinker	1117 (43)	1899 (49)	2150 (62)	
Heavy drinker	153 (6)	254 (7)	268 (8)	
**Access to care**				
Insurance coverage				<.0001
Yes	1686 (64)	3038 (79)	3265 (95)	
No	931 (36)	817 (21)	188 (5)	
**Health status**				
Self-rated health				<.0001
Good	1753 (67)	3085 (80)	3155 (91)	
Fair/poor	864 (33)	770 (20)	298 (9)	
Disabilities, Yes (vs. No)				
ADL	293 (11)	225 (6)	126 (4)	<.0001
IADL	402 (15)	309 (8)	189 (5)	<.0001
LSA	292 (11)	217 (6)	118 (3)	<.0001
LEM	397 (15)	352 (9)	160 (5)	<.0001
GPA	552 (21)	552 (14)	374 (11)	<.0001
**Biological**				
CRP, mg/L				<.0001
<1	610 (23)	945 (25)	1025 (30)	
1–3	826 (32)	1338 (35)	1179 (34)	
>3–10	826 (32)	1154 (30)	929 (27)	
>10	355 (14)	418 (11)	320 (9)	

Abbreviations: ADL: Activities of daily living; BMI: Body mass index; CRP: C-reactive protein; CVD: Cardiovascular disease; GPA: General physical activities; HEI: Healthy eating index; IADL: Instrumental activities of daily living; LEM: Lower extremity mobility; LSA: Leisure and social activities; PIR: Poverty-income ratio.

Data are N (%) unless otherwise indicated. ^a^ χ^2^ (chi-square) analysis was used to compare frequencies and ANOVA was used to compare mean age between groups.

**Table 2:** Mortality rate and hazard ratios of mortality for individual mediators.

		All-cause mortality			CVD/diabetes mortality		
Individual mediators	Person-years	Mortality rate^a^	HR^b^ (95% CI)		Mortality rate^a^	HR^b^ (95% CI)	
**Health behaviors**							
Diet (HEI-2005 quintiles)							
Q1	124462	10.32	1.46 (1.02, 2.10)		3.57	1.28 (0.77, 2.13)	
Q2	117036	8.72	1.13 (0.74, 1.72)		2.46	0.94 (0.44, 1.99)	
Q3	116052	9.31	1.06 (0.72, 1.54)		4.55	1.51 (0.83, 2.74)	
Q4	111506	12.81	1.19 (0.88, 1.63)		5.27	1.29 (0.70, 2.37)	
Q5	104883	11.90	1.00		4.81	1.00	
MET-hours/week of leisure-time physical activity							
Inactive (0)	238696	15.23	1.84 (1.34, 2.53)		6.43	3.29 (1.64, 6.60)	
Somewhat active (>0 to <9)	169797	10.32	1.26 (0.88, 1.81)		3.89	2.04 (1.02, 4.05)	
Active (≥9)	165446	4.06	1.00		0.94	1.00	
Smoking							
Yes	124304	10.23	1.96 (1.29, 2.97)		3.57	2.19 (1.22, 3.93)	
No	449635	10.65	1.00		4.24	1.00	
Alcohol use							
Non-drinker	235655	14.46	1.31 (0.97, 1.77)		6.01	1.46 (0.96, 2.22)	
Moderate drinker	298845	7.71	1.00		2.73	1.00	
Heavy drinker	39439	8.82	1.62 (0.96, 2.76)		3.04	1.30 (0.62, 2.74)	
BMI, kg/m^2^							
≥40.0	27190	7.06	1.42 (0.85, 2.37)		2.21	2.12 (0.74, 6.09)	
30.0–39.9	156413	8.98	0.96 (0.68, 1.33)		3.07	0.69 (0.40, 1.20)	
25.0–29.9	208468	10.48	0.69 (0.49, 0.96)		4.37	0.72 (0.44, 1.19)	
18.5–24.9	173521	12.38	1.00		4.84	1.00	
<18.5	8347	15.81	1.42 (0.52, 3.89)		7.19	1.03 (0.29, 3.63)	
**Access to medical care**							
Insurance coverage							
Yes	460668	12.27	1.00		4.79	1.00	
No	113271	3.60	1.18 (0.70, 2.00)		1.27	1.95 (0.79, 4.80)	
**Health status**							
Self-rated health							
Good	464110	7.60	1.00		2.71	1.00	
Fair/poor	109829	23.05	2.61 (2.03, 3.36)		9.94	2.50 (1.63, 3.84)	
Disabilities, Yes (vs. No)							
ADL	33819	34.77	1.19 (0.84, 1.68)		15.97	1.54 (0.89, 2.68)	
IADL	48654	30.83	1.27 (0.83, 1.95)		14.06	1.49 (0.84, 2.65)	
LSA	33591	36.44	1.17 (0.84, 1.62)		15.72	1.17 (0.74, 1.84)	
LEM	48975	37.49	1.42 (0.91, 2.23)		15.44	0.90 (0.40, 2.06)	
GPA	79675	28.77	1.35 (0.89, 2.05)		12.05	1.48 (0.69, 3.19)	
**Biological**							
CRP, mg/L							
<1	148784	5.00	1.00		1.94	1.00	
1–3	191555	10.40	1.55 (1.07, 2.23)		4.32	1.42 (0.70, 2.89)	
>3–10	170630	13.43	2.05 (1.47, 2.86)		5.34	1.67 (0.77, 3.63)	
>10	62970	16.39	2.90 (1.95, 4.33)		5.15	1.71 (0.80, 3.66)	

Abbreviations: ADL: Activities of daily living; BMI: Body mass index; CRP: C-reactive protein; CVD: Cardiovascular disease; GPA: General physical activities; HEI: Healthy eating index; IADL: Instrumental activities of daily living; LEM: Lower extremity mobility; LSA: Leisure and social activities; PIR: Poverty-income ratio.

a The mortality rates are per 1,000 person-years;

b Hazard ratio in separate models for each mediator, controlled for income, age, sex, education and race/ethnicity.

**Table 3:** Mediating effect of several factors on all-cause mortality and CVD/diabetes mortality among low- and intermediate-income groups.

	Low income		Intermediate income	
	HR (95% CI)	% Attenuation^b^(95% CI)	HR (95% CI)	% Attenuation^b^(95% CI)
**All-cause mortality**				
Model I a	1.98 (1.37, 2.85)	–	1.50 (1.10, 2.03)	-
+Health behaviors (5 measures)	1.61 (1.11, 2.33)	30 (15, 64)	1.33 (0.99, 1.79)	30 (11, 131)
Diet	1.93 (1.33, 2.78)	4 (−1, 13)	1.48 (1.08, 2.01)	4 (−2, 20)
Physical activity	1.78 (1.24, 2.57)	15 (7, 34)	1.43 (1.06, 1.92)	12 (4, 48)
Smoking	1.85 (1.28, 2.67)	10 (4, 24)	1.44 (1.06, 1.94)	10 (3, 43)
Alcohol	1.90 (1.32, 2.72)	6 (−1, 19)	1.46 (1.08, 1.96)	7 (−2, 35)
BMI	1.94 (1.35, 2.79)	1 (−2, 12)	1.47 (1.09, 1.99)	4 (−2, 19)
+Health insurance	1.93 (1.32, 2.83)	3 (−6, 24)	1.48 (1.09, 2.01)	3 (−7, 30)
+Health status (2 measures)	1.51 (1.06, 2.17)	39 (20, 76)	1.38 (1.04, 1.84)	20 (4, 70)
Self-rated health	1.68 (1.16, 2.42)	24 (11, 50)	1.44 (1.07, 1.95)	9 (−3, 39)
Disabilities	1.63 (1.14, 2.33)	28 (13, 55)	1.42 (1.07, 1.89)	13 (1, 43)
+CRP	1.94 (1.35, 2.79)	3 (−4, 12)	1.50 (1.11, 2.03)	−1 (−15, 10)
Full model	1.33 (0.91, 1.94)	58 (28, 126)	1.27 (0.95, 1.71)	41 (11, 170)
**CVD/diabetes mortality**				
Model II a	3.68 (1.64, 8.27)	–	2.00 (1.11, 3.61)	–
+Health behaviors (5 measures)	2.79 (1.32, 5.92)	21 (10, 39)	1.71 (0.98, 2.98)	22 (7, 90)
Diet	3.66 (1.61,8.30)	0 (−4, 6)	1.98 (1.08, 3.62)	1 (−6, 14)
Physical activity	3.10 (1.44,6.72)	13 (6, 25)	1.84 (1.05, 3.24)	12 (3, 48)
Smoking	3.42 (1.53,7.68)	5 (1, 13)	1.91 (1.08, 3.37)	7 (1, 32)
Alcohol	3.41 (1.56,7.46)	6 (0, 15)	1.90 (1.07, 3.38)	7 (−1, 31)
BMI	3.64(1.60, 8.26)	1 (−3, 6)	1.99 (1.09, 3.64)	0 (−7, 11)
+Health insurance	3.41 (1.44, 8.07)	6 (−2, 25)	1.92 (1.05, 3.52)	6 (−4, 34)
+Health status (2 measures)	2.92 (1.34, 6.40)	18 (7, 34)	1.90 (1.08, 3.34)	8 (−7, 44)
Self-rated health	3.17 (1.44, 6.98)	11 (4, 25)	1.94 (1.09, 3.44)	5 (−3, 25)
Disabilities	3.13 (1.41, 6.93)	13 (4, 29)	1.94 (1.08, 3.48)	4 (−8, 27)
+CRP	3.65 (1.62,8.25)	1 (−2, 5)	1.99 (1.10, 3.61)	0 (−6, 8)
Full model	2.33 (1.05, 5.16)	35 (16, 68)	1.61 (0.91, 2.87)	31 (4, 150)

a age, sex, education, and race/ethnicity adjusted.

b Calculated as: (β _Model I –_ β _Model I+mediator(s)_) × 100/β _Model I_ for all-cause mortality (β _Model II -_ β _Model II+mediator(s)_) × 100/β _Model II_ for CVD/diabetes mortality.

## Results

Of the 31,126 individuals who participated in the 1999–2004 NHANES, 14213 (46%) were aged 20 years or older and attended the mobile examination center. Of these, 9925 were included in this analysis after listwise deletion of those with missing data on mortality (n = 20), income (n = 1252), or other variables (n = 3016); missing data on each variable was less than 10%. Compared to the study sample, those who were excluded were on average 4.0 years older (95% CI: 2.9, 5.1), more likely to have low-income (38% vs. 26%), be non-Hispanic Black (23% vs. 18%), and had higher mortality rates for all-cause mortality (19.48 vs. 10.56 per 1000 person-years) and CVD/diabetes mortality (7.97 vs. 4.10 per 1000 person-years) (each *P*<0.05). Among the study sample, 26% had low income and 35% had high income. Follow-up was on average 4.8 years (median 4.8, range 0.1–7.8). During the follow-up, 505 persons died, including 196 deaths (39.0%) due to CVD or diabetes.

**Figure 2:**
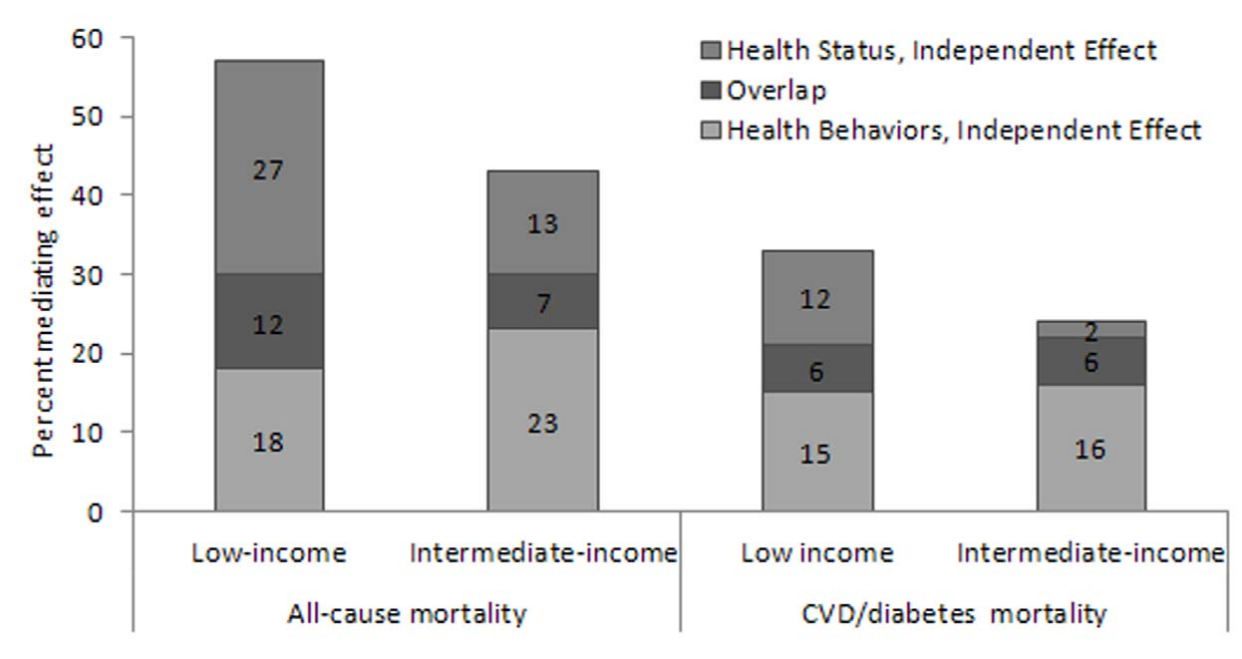
Overlap Between Health Behaviors and Health Status. Example of calculation for all-cause mortality, low-income group: Mediating effect of Health Behaviors and Health Status = 57% Mediating effect of Health Behaviors = 30%; Mediating effect of Health Status = 39% Combined effect = 30%+39% = 69% Overlap = 69% –57% = 12% Health Behaviors, independent Effect = 30%–12% = 18% Health Status, Independent Effect = 39%–12% = 27%.

**Table 4:** Mortality Rates and Hazard Ratios of Mortality for Income and Education, by Gender and Race/ethnicity.

	Total	Gender		Race/ethnicity		
		Women	Men	Non-Hispanic Black	Mexican American	Non-Hispanic White
	HR (95% CI)	HR (95% CI)	HR (95% CI)	HR (95% CI)	HR (95% CI)	HR (95% CI)
Person-years	47828.25	24914.92	22913.33	8442.17	11324.58	24538.25
**All-cause mortality**						
N. of deaths	505	201	304	98	79	309
Mortality rate	10.56	8.07	13.27	11.61	6.98	12.59
Income						
Low, PIR ≤1.30	1.98 (1.37, 2.85)	1.81 (0.97, 3.37)	2.01 (1.23, 3.30)	4.53 (2.10, 9.78)	1.22 (0.28, 5.32)	1.98 (1.30, 3.00)
Medium, 1.3<PIR<3.5	1.50 (1.10, 2.03)	1.25 (0.71, 2.20)	1.68 (1.22, 2.33)	3.32 (1.58, 6.96)	0.61 (0.18, 2.14)	1.46 (1.04, 2.04)
High, PIR ≥3.50	1.00	1.00	1.00	1.00	1.00	1.00
Education						
<High school	1.68 (1.12, 2.51)	2.11 (1.04, 4.27)	1.53 (0.87, 2.66)	1.22 (0.59, 2.53)	0.35 (0.07, 1.62)	1.92 (1.30, 2.82)
High school/some college	1.60 (1.09, 2.34)	2.05 (0.98, 4.26)	1.44 (0.86, 2.42)	1.30 (0.59, 2.85)	0.37 (0.10, 1.36)	1.78 (1.23, 2.58)
≥ College graduate	1.00	1.00	1.00	1.00	1.00	1.00
**CVD/diabetes mortality**						
N. of deaths	196	80	116	34	34	120
Mortality rate	4.10	3.21	5.06	4.03	3.00	4.89
Income						
Low, PIR ≤1.30	3.68 (1.64, 8.27)	1.98 (0.82, 4.77)	5.43 (1.95, 15.10)	22.04 (2.58, 188.50)	1.47 (0.05, 47.77)	3.29 (1.39, 7.76)
Medium, 1.3<PIR<3.5	2.00 (1.11, 3.61)	0.96 (0.52, 1.78)	3.34 (1.46, 7.64)	17.70 (2.27, 137.76)	0.49 (0.03, 9.49)	1.62 (0.87, 3.01)
High, PIR ≥3.50	1.00	1.00	1.00	1.00	1.00	1.00
Education						
<High school	0.97 (0.43, 2.16)	1.29 (0.37, 4.45)	0.91 (0.38, 2.15)	2.19 (0.22, 22.41)	†	1.11 (0.49, 2.50)
High school/some college	0.91 (0.46, 1.79)	1.59 (0.53, 4.73)	0.63 (0.26, 1.51)	2.83 (0.32, 25.41)	‡	0.98 (0.48, 2.00)
≥ College graduate	1.00	1.00	1.00	1.00		1.00

† 1370320 (67885.83, 27660815);

‡ 1773536 (695560.1, 4522152).

The unadjusted all-cause mortality rates per 1000 person-years among the low-, intermediate-, and high-income groups were 13.61, 12.85, and 5.77, respectively. The unadjusted CVD/diabetes mortality rates per 1000 person-years among the three income groups were 6.00, 4.97 and 1.72, respectively. After controlling for age, sex, education and race/ethnicity, the risk of all-cause mortality was higher in the low-income (HR: 1.98, 95% CI: 1.37, 2.85) and the intermediate-income (HR: 1.50, 95% CI: 1.10, 2.03), compared to the high-income group. The adjusted risk of CVD/diabetes mortality was 3.68 (95% CI: 1.64, 8.27) in the low-income and 2.00 (95% CI: 1.11, 3.61) in the intermediate-income group compared to the high-income group.

Table 1 shows that the distribution of all potential mediators differed across the income groups. Compared to the high-income group, people with low-income were on average 3.4 years younger, were less likely to be non-Hispanic White, college graduates, physically active, consume alcohol moderately, and have good diet, health insurance coverage, “good” self-rated health, and low levels of CRP. In addition, individuals of low income were more likely to smoke, be obese, have CVD/diabetes morbidity, and disabilities than those with higher incomes.

As shown in Table 2, the potential mediators each were associated with increased risk of mortality.

Table 3 shows that the association between income and either all-cause or CVD/diabetes mortality was attenuated to varying extents by individual mediators and substantially by all mediators combined. The combination of all mediators attenuated 58% of all-cause mortality in the low-income group, where there was no longer direct influence of income. For all-cause mortality, the largest mediating effect in the low-income group was for health status (39%), followed by health behaviors (30%), CRP (3%) and health insurance (3%). Among the measures of health status, disabilities, and among the health behaviors, physical activity showed the largest mediating effects. For the intermediate income group, the pattern of mediation was generally similar to the low-income group except that health status attenuated only 20% of the income disparity, resulting in an overall lower extent of mediation (41%) than for the low-income group.

Similarly, for CVD/diabetes mortality, health behaviors and health status had the largest mediating effects, 21% and 18%, respectively, while health insurance and CRP each accounted for only a small portion of the low-income disparity. Of all mediators, physical activity and disabilities accounted for the largest proportion of the effect of income on mortality. Overall, the full model attenuated 35% of the CVD/diabetes mortality in the low-income group and 31% in the intermediate income group.

As depicted in Figure 2, after subtracting the overlap between health behaviors and health status, the relative contributions of health behaviors was 15–23% across both income groups and types of mortality. The independent contribution of health status to either all-cause or CVD/diabetes mortality was largest among the low-income group.

When hazard ratios were estimated for education, the results showed that low-education was associated with all-cause mortality, but not with CVD/diabetes mortality (Table 4). In stratified analyses, low education was associated with all-cause mortality among women, not among men, and among non-Hispanic Whites, not among other race/ethnicity groups. In addition, low income was associated with either all-cause mortality or CVD/diabetes mortality, with stronger associations among men and among non-Hispanic Blacks.

The results of the sensitivity analysis excluding participants who were unemployed because of health reasons (N = 249), were similar to the original models; the combination of the four pathways attenuated 60% of the association between low income and all-cause mortality and 37% of that of CVD/diabetes mortality. In addition, results of the age stratified models showed that among individuals aged 20–44 years, unlike the older age groups, income was not associated with mortality. Moreover, in the age groups 45–64 and ≥65 years, respectively, the combination of the four pathways attenuated 66% and 48% of the association between income and all-cause mortality and 44% and 41% of that of CVD/diabetes mortality.

## Discussion

This nationally representative study of US adults assessed the mediating effects of health behaviors, health insurance coverage, health status and CRP on the association between income and all-cause and CVD/diabetes mortality. The combination of the four groups of factors accounted for the vast majority of why persons with low- and those with intermediate incomes were more likely to die compared to those with higher incomes. The results provide new insight into the pathways that mediate income disparities in health by demonstrating that besides unhealthy behaviors, poor health status also plays an important role in income disparities in health.

Consistent with previous studies, health behaviors accounted for only part of the reason for socioeconomic disparity in mortality. In particular, health behaviors attenuated 30% of all-cause mortality in this study compared to 12% in a previous US study [2] and 42% in a European study, with single assessment, and 72% with repeated assessments [9]. The previous US study may have underestimated the impact of health behaviors partly because that study did not include diet as a component of health behaviors. Of the health behaviors assessed in this study, physical inactivity followed by smoking had the largest mediating effect on the association between income and either all-cause or CVD/diabetes mortality. Sedentary lifestyle and smoking, which are risk factors for all-cause and CVD mortality, were about twice as prevalent among the low-income as high-income group. Available evidence suggests that both individual and environmental influences should be addressed in strategies to improve sedentary lifestyle and smoking behaviors in people of low socioeconomic status [32], [33]. Notably, BMI, diet and alcohol had only a minor contribution in the income-mortality association, likely because of the small variation in these factors among the income groups, consistent with some previous studies [34], [35].

A more complete picture of socioeconomic disparities in mortality involves several other factors besides health behaviors, such as access to medical care, biological factors, and health status. Among these factors, poor health status was an important mediator in this study. One possible explanation is that worse health status is a consequence of unhealthy behaviors. However, a longitudinal nationally representative US study showed that unhealthy behaviors, including smoking, physical inactivity, alcohol use, and overweight, accounted for only a small proportion of the subsequent socioeconomic disparities in health status, measured by physical functioning and self-rated health [36]. Likewise, the finding of a small amount of overlap between health behaviors and health status, suggests that health status and health behaviors, although interrelated, are at least partly independent mediating pathways. An alternative explanation is that a worse health status may reflect the influence of adverse social and physical conditions, such as stressful life conditions and psychosocial factors [37]. The impact of psychosocial and environmental stressors on physiological processes can be mediated through several mechanisms, such as adverse effects on immunity [38] and accelerating cellular mechanisms of aging [39].

Contrary to health behaviors and health status, access to health care and biological factors had negligible contributions to income related mortality. Given that the proportion of people who were uninsured was greater in the low-income group, the reason for the small mediating effect may be partly related to the income related differences in types of insurance. In the US, public insurance has been associated with a greater risk of mortality than private insurance [40]. Regarding CRP, the finding of low explanatory power is in accordance with a previous study that showed CRP alone reflects only a small amount of the biological risk associated with low socioeconomic status [10]. Although several studies, including this study, indicate that CRP is a strong predictor of mortality, controversy continues about its direct causal effect [41]. Whatever the mechanism, the association between CRP and mortality seems to be largely independent of the pathways linking low income to mortality. Future research should clarify the possible role of other biological markers.

This study has some limitations. First, the models did not include some potential mediators such as environmental factors. However, it is likely that these factors exert their effect through the more proximate mediators included in the statistical models. Second, mediators were measured only at one time point. Because some of the mediators may change differentially over time [9], the findings need to be verified in future studies that include repeated measurements. Third, the measures were mainly self-reported, without objective measures of health status, and subject to response bias. Because of the general tendency of underreporting unhealthy behaviors [42] and chronic illnesses [43], the impact of both health behaviors and health status might be underestimated in this study. Forth, only one marker of systemic inflammation, i.e., CRP, was assessed. Although CRP is the most commonly used marker of inflammation, inclusion of additional markers, such as fibrinogen and IL-6, would have increased the accuracy of the assessment of inflammation. However, the additional markers were not available for our study participants. Finally, individuals who were excluded had generally lower health status, poorer health behaviors and a higher mortality rate than those included in the study. However, although the possibility of non-response bias cannot be completely eliminated, it is unlikely that non-response bias had a major effect on the estimates, since the distributions of mediators among the income groups were similar between included and excluded samples. Despite these limitations, strengths of this nationally representative study include quantitative estimates of various mediating pathways that could be used to prioritize implementation of interventions aimed at reducing income disparities in mortality.

In summary, this study supports the findings from previous studies that health behaviors are important mechanisms mediating income disparities in risk of death. The present study extends previous findings by showing that income disparities in mortality are also the result of poor health status. To reduce mortality in low income people, more emphasis may be needed in improving health status. Future studies should address behavioral modification, as well as possible strategies to improve health status in low-income people.

## References

[1] Singh GK (2003) Area deprivation and widening inequalities in US mortality, 1969–1998. Am J Public Health 93: 1137–43. 12835199 10.2105/ajph.93.7.1137 PMC1447923

[2] Lantz PM, House JS, Lepkowski JM, Williams DR, Mero RP, et al (1998) Socioeconomic factors, health behaviors, and mortality. JAMA 279: 1703–8. 9624022 10.1001/jama.279.21.1703

[3] Adler NE, Newman K (2002) Socioeconomic disparities in health: Pathways and policies. Health Aff 21: 60–76. 10.1377/hlthaff.21.2.60 11900187

[4] Lynch JW, Smith GD, Kaplan GA, House JS (2000) Income inequality and mortality: Importance to health of individual income, psychosocial environment, or material conditions. BMJ 320: 1200–1204. 10784551 10.1136/bmj.320.7243.1200 PMC1127589

[5] Marmot M, Wilkinson RG (2001) Psychosocial and material pathways in the relation between income and health: A response to Lynch et al. BMJ 322: 1233–1236. 11358781 10.1136/bmj.322.7296.1233 PMC1120336

[6] Macleod J, Smith GD, Metcalfe C, Hart C (2005) Is subjective social status a more important determinant of health than objective social status? Evidence from a prospective observational study of Scottish men. Soc Sci Med 61: 1916–1929. 15916842 10.1016/j.socscimed.2005.04.009

[7] Wilkinson RG, Pickett KE (2009) Income inequality and social dysfunction. Annu Rev Sociol 35: 493–511.

[8] McEwen BS, Seeman T (1999) Protective and damaging effects of mediators of stress: elaborating and testing the concepts of allostasis and allostatic load. Annals of the New York Academy of Sciences 896: 30–47. 10681886 10.1111/j.1749-6632.1999.tb08103.x

[9] Stringhini S, Sabia S, Shipley M, Brunner E, Nabi H, et al (2010) Association of socioeconomic position with health behaviors and mortality. JAMA 303: 1159–66. 20332401 10.1001/jama.2010.297 PMC2918905

[10] Seeman TE, Crimmins E, Huang M-H, Singer B, Bucur A, et al (2004) Cumulative biological risk and socioeconomic differences in mortality: MacArthur Studies of Successful Aging. Soc Sci Med 58: 1985–97. 15020014 10.1016/S0277-9536(03)00402-7

[11] U.S. Department of Health and Human Services. Healthy People 2020 topics and objectives. Social Determinants of Health. Available: http://healthypeople.gov/2020/topicsobjectives2020/overview.aspx?topicid=39. Accessed 16 Nov 2011.

[12] Feinstein JS (1993) The Relationship between Socioeconomic Status and Health: A Review of the Literature. The Milbank Quarterly 71: 279–322. 8510603

[13] U.S. Center for Disease Control and Prevention. Health, United States, 2010, With Special Feature on Death and Dying. Available: http://www.cdc.gov/nchs/data/hus/hus10.pdf#highlights. Accessed 26 Oct 2011.

[14] Franks P, Gold MR, Fiscella K (2003) Sociodemographics, self-rated health, and mortality in the US. Soc Sci Med 56: 2505–2514. 12742613 10.1016/s0277-9536(02)00281-2

[15] Alley DE, Seeman TE, Ki Kim J, Karlamangla A, Hu P, et al (2006) Socioeconomic status and C-reactive protein levels in the US population: NHANES IV. Brain Behav Immun 20: 498–504. 16330181 10.1016/j.bbi.2005.10.003

[16] Seeman T, Merkin SS, Crimmins E, Koretz B, Charette S, et al (2008) Education, income and ethnic differences in cumulative biological risk profiles in a national sample of US adults: NHANES III (1988–1994). Soc Sci Med 66: 72–87. 17920177 10.1016/j.socscimed.2007.08.027 PMC2180425

[17] Wagstaff A, van Doorslaer E (2000) Income Inequality and Health: What does the literature tell us? Annu Rev Public Health 21: 543–567. 10884964 10.1146/annurev.publhealth.21.1.543

[18] Lynch JW, Kaplan GA, Pamuk ER, Cohen RD, Heck KE, et al (1998) Income inequality and mortality in metropolitan areas of the United States. Am J Public Health 88: 1074–1080. 9663157 10.2105/ajph.88.7.1074 PMC1508263

[19] Fiscella K, Franks P (2000) Individual income, income inequality, health, and mortality: What are the relationships? Health Serv Res 35: 307–318. 10778817 PMC1089103

[20] Centers for Disease Control and Prevention (CDC) National Center for Health Statistics (NCHS). National Health and Nutrition Examination Survey Data. Hyattsville, MD: US Department of Health and Human Service, Centers for Disease Control and Prevention, NHANES 1999–2000, 2001–2002, 2003–2004. Available: http://www.cdc.gov/nchs/nhanes.htm. Accessed 9 May 2011.

[21] Wong MD, Shapiro MF, Boscardin WJ, Ettner SL (2002) Contribution of major diseases to disparities in mortality. N Engl J Med 347: 1585–92. 12432046 10.1056/NEJMsa012979

[22] Analytic and reporting guidelines. The Third National Health and Nutrition Examination Survey, NHANES III (1988–94). Available: http://www.cdc.gov/nchs/data/nhanes/nhanes3/nh3gui.pdf. Accessed 9 May 2011.

[23] U.S. Department of Health and Human Services and US Department of Agriculture (2005) Dietary Guidelines for Americans, 2005. Washington, DC: US Government Printing Office.

[24] George SM, Neuhouser ML, Mayne ST, Irwin ML, Albanes D, et al (2010) Postdiagnosis diet quality is inversely related to a biomarker of inflammation among breast cancer survivors. Cancer Epidemiol Biomarkers Prev 19: 2220–8. 20716617 10.1158/1055-9965.EPI-10-0464 PMC3077799

[25] Avlund K (1997) Methodological challenges in measurements of functional ability in gerontological research. A review. Aging Clin Exp Res 9: 164–74. 10.1007/BF03340145 9258374

[26] Duncan PW, Samsa GP, Weinberger M, Goldstein LB, Bonito A, et al (1997) Health status of individuals with mild stroke. Stroke 28: 740–5. 9099189 10.1161/01.str.28.4.740

[27] Chen H, Guo X (2008) Obesity and functional disability in elderly Americans. J Am Geriatr Soc 56: 689–94. 18266843 10.1111/j.1532-5415.2007.01624.x PMC2391089

[28] Centers for Disease Control and Prevention (CDC), National Center for Health Statistics (NCHS). National Health and Nutrition Examination Laboratory Protocol NHANES 1999–2000, 2001–2002, 2003–2004. Available: www.cdc.gov/nchs/nhanes.htm. Accessed 9 May 2011.

[29] Pearson TA, Mensah GA, Alexander RW, Anderson JL, Cannon RO, et al (2003) Markers of inflammation and cardiovascular disease: Application to clinical and public health practice: a statement for healthcare professionals from the Centers for Disease Control and Prevention and the American Heart Association. Circulation 107: 499–511. 12551878 10.1161/01.cir.0000052939.59093.45

[30] Efron B, Tibshirani RJ (1993) An introduction to the bootstrap. Chapter 14, pp 178–201. New York: Chapman & Hall.

[31] Linder SH, Sexton K (2011) Conceptual models for cumulative risk assessment. Am J Public Health101: S74–S81. 10.2105/AJPH.2011.300318 PMC3222476 22021317

[32] Fisher EB, Auslander WF, Munro JF, Arfken CL, Brownson RC, et al (1998) Neighbors for a smoke free north side: evaluation of a community organization approach to promoting smoking cessation among African Americans. Am J Public Health 88: 1658–63. 9807532 10.2105/ajph.88.11.1658 PMC1508568

[33] Giles-Corti B, Donovan RJ (2002) The relative influence of individual, social and physical environment determinants of physical activity. Soc Sci Med 54: 1793–812. 12113436 10.1016/s0277-9536(01)00150-2

[34] Chang VW, Lauderdale DS (2005) Income disparities in body mass index and obesity in the United States, 1971–2002. Arch Intern Med 165: 2122–8. 16217002 10.1001/archinte.165.18.2122

[35] Lantz PM, Golberstein E, House JS, Morenoff J (2010) Socioeconomic and behavioral risk factors for mortality in a national 19-year prospective study of U.S. adults. Soc Sci Med 70: 1558–66. 20226579 10.1016/j.socscimed.2010.02.003 PMC3337768

[36] Lantz PM, Lynch JW, House JS, Lepkowski JM, Mero RP, et al (2001) Socioeconomic disparities in health change in a longitudinal study of US adults: the role of health-risk behaviors. Soc Sci Med 53: 29–40. 11380160 10.1016/s0277-9536(00)00319-1

[37] Schulz AJ, Kannan S, Dvonch JT, Israel BA, Allen Iii A, et al (2005) Social and physical environments and disparities in risk for cardiovascular disease: The healthy environments partnership conceptual model. Environ Health Perspect 113: 1817–25. 16330371 10.1289/ehp.7913 PMC1314928

[38] Black PH, Garbutt LD (2002) Stress, inflammation and cardiovascular disease. J Psychosom Res 52: 1–23. 11801260 10.1016/s0022-3999(01)00302-6

[39] Epel ES, Blackburn EH, Lin J, Dhabhar FS, Adler NE, et al (2004) Accelerated telomere shortening in response to life stress. Proc Nati Acad Sci USA 101: 17312–5. 10.1073/pnas.0407162101 PMC534658 15574496

[40] Rask K, O’Malley E, Druss B (2009) Impact of socioeconomic, behavioral and clinical risk factors on mortality. J Public Health 31: 231–8. 10.1093/pubmed/fdp015 19279019

[41] The Emerging Risk Factors Collaboration (2010) C-reactive protein concentration and risk of coronary heart disease, stroke, and mortality: an individual participant meta-analysis. Lancet 375: 132–40. 20031199 10.1016/S0140-6736(09)61717-7 PMC3162187

[42] Shephard RJ (2003) Limits to the measurement of habitual physical activity by questionnaires. Br J Sports Med 37: 197–206. 12782543 10.1136/bjsm.37.3.197 PMC1724653

[43] Mackenbach JP, Looman CW, van der Meer JB (1996) Differences in the misreporting of chronic conditions, by level of education: the effect on inequalities in prevalence rates. Am J Public Health 86: 706–11. 8629723 10.2105/ajph.86.5.706 PMC1380480

